# Cilostazol disrupts TLR-4, Akt/GSK-3β/CREB, and IL-6/JAK-2/STAT-3/SOCS-3 crosstalk in a rat model of Huntington's disease

**DOI:** 10.1371/journal.pone.0203837

**Published:** 2018-09-27

**Authors:** Hanan El-Abhar, Mai A. Abd El Fattah, Walaa Wadie, Dalia M. El-Tanbouly

**Affiliations:** Department of Pharmacology and Toxicology, Faculty of Pharmacy, Cairo University, Cairo, Egypt; University of Hong Kong, HONG KONG

## Abstract

Countless neurodegenerative diseases are associated with perverse multiple targets of cyclic nucleotide signalling, hastening neuronal death. Cilostazol, a phosphodiesterase-III inhibitor, exerts neuroprotective effects against sundry models of neurotoxicity, however, its role against Huntington’s disease (HD) has not yet been tackled. Hence, its modulatory effect on several signalling pathways using the 3-nitropropionic acid (3-NP) model was conducted. Animals were injected with 3-NP (10 mg/kg/day, i.p) for two successive weeks with or without the administration of cilostazol (100 mg/kg/day, p.o.). Contrary to the 3-NP effects, cilostazol largely preserved striatal dopaminergic neurons, improved motor coordination, and enhanced the immunohistochemical reaction of tyrosine hydroxylase enzyme. The anti-inflammatory effect of cilostazol was documented by the pronounced reduction of the toll like receptor-4 (TLR-4) protein expression and the inflammatory cytokine IL-6, but with a marked elevation in IL-10 striatal contents. As a consequence, cilostazol reduced IL-6 downstream signal, where it promoted the level of suppressor of cytokine signalling 3 (SOCS3), while abated the phosphorylation of Janus Kinase 2 (JAK-2) and Signal transducers and activators of transcription 3 (STAT-3). Phosphorylation of the protein kinase B/glycogen synthase kinase-3β/cAMP response element binding protein (Akt/GSK-3β/CREB) cue is another signalling pathway that was modulated by cilostazol to further signify its anti-inflammatory and antiapoptotic capacities. The latter was associated with a reduction in the caspase-3 expression assessed by immunohistochemical assay. In conclusion the present study provided a new insight into the possible mechanisms by which cilostazol possesses neuroprotective properties. These intersecting mechanisms involve the interference between TLR-4, IL-6-IL-10/JAK-2/STAT-3/SOCS-3, and Akt/GSK-3β/CREB signalling pathways.

## Introduction

One of the progressive neurodegenerative disorders is the Huntington’s disease (HD), which is an autosomal inherited disease that targets mainly the striatum. [1]. Psychiatric disturbances, cognitive debility, as well as waning of motor function are the triad that signifies the clinical pathological changes in HD [2]. The 3-nitropropionic acid (3-NP) is an irreversible inhibitor of succinate dehydrogenase (SDH), a mitochondrial complex II enzyme, responsible for the oxidation of succinate to fumarate in the Kreb’s cycle. In turn, it hinders the electron transport in the oxidative phosphorylation flow and impairs mitochondrial function, hence, resulting in a decrease in ATP levels and oxidative burst, events that integrate to cause neuronal injury [3,4]. 3-NP closely simulates many of the neuropathological and behavioral features of HD; hence, 3-NP treated rodents provided a relevant model to examine possible treatments for HD [5]. More interestingly, repeated administration of 3-NP at low doses provokes motor alteration from hyperactivity to hypoactivity depicting the progression from chorea to a parkinsonian-like syndrome in human[6].

The level of cyclic adenosine monophosphate (cAMP) decreases in various neuropathological conditions [7–9] and aggravation of neuroinflammatory processes in neurodegenerative diseases was robustly correlated with aberrant cAMP signalling, possibly originating from abnormal PDEs expression [10]. The study of cyclic nucleotide signalling in the last decades has revealed a stunning complexity and may exploit several cellular pathways [11].

Cilostazol, a type III phosphodiesterase inhibitor, increases intracellular cAMP levels; the drug was approved by the Food and Drug Administration for the treatment of intermittent claudication, besides, its principal actions including inhibition of platelet aggregation, antithrombotic action in cerebral ischemia, and vasodilation mediated by the increased cAMP levels [12]. Cilostazol has been shown in a multicenter, randomized, and double-blind clinical trial to provide a considerable risk reduction in patients with recurrent cerebral infarction [13]. Several *in* vivo studies revealed that cilostazol may be a powerful candidate to protect against brain lesions and cognitive impairment associated with chronic cerebral hypoperfusion and focal cerebral ischemia [14–19].

Based on the previous background, the present study was conducted to investigate the potential of cilostazol on the striatal neuropathological as well as behavioural derangements induced by 3-NP in rats. It also extended to explore some of the possibly integrating signalling cues that could offer neuroprotection against 3-NP model.

## Material and methods

### Ethics statement

This study was carried out in strict accordance with the recommendations in the Guide for the Care and Use of Laboratory Animals of the National Institutes of Health (NIH publication No.85-23, 1996). The protocol was reviewed and approved by the Ethics Research Committee of Faculty of Pharmacy, Cairo University (PT: 2052). All efforts were exerted to minimize animal suffering during the experimental period. Where, the duration of the experiment was as short as possible, the number of animals was kept to a minimum and all animals were sacrificed by decapitation under light anaesthesia.

### Animals

Adult male Wistar rats (150 ± 20 g) were obtained from National Research Centre (NRC, Giza, Egypt). Animals were allowed to acclimatize in the animal facility of Faculty of Pharmacy (Cairo University) for one week prior to starting any experimental procedure. Rats were allowed free access to standard chow pellet and water *ad libitum* and were kept under controlled environmental conditions (constant temperature of 23±2°C, humidity of 60±10%, and light/dark (12/12 h) cycle with lights on at 6:00 am). All behavioural tests were carried out in a sound isolated laboratory.

### Experimental design

Rats were divided randomly into 4 groups (n = 12); group 1 represents the normal control group and animals received saline, while group 2 depicts cilostazol (Otsuka pharmaceutical Co. S.A.E, Cairo, Egypt) normal treated group and rats were gavaged cilostazol orally (100 mg/kg/day [20]**).** In groups 3 and 4, rats were injected daily with 3-NP (Sigma-Aldrich, MO, US; 10 mg/kg, i.p; [21]**)** without and with cilostazol for 14 days to serve as the HD model and the cilostazol treated groups, respectively. On the last day of the experiment, rats were evaluated for behavioural tests, then sacrificed and the striata were collected for biochemical, western blot, and immunohistochemical analysis.

### Behavioural tests

Twenty-four hours after the last dose of treatments, rats were screened for motor performance using manual gait analysis, pole, and cylinder tests.

#### Manual gait analysis

This experiment was adopted from previous 6-hydroxydopamine-lesioned rat model [22,23] to assess the 3-NP-induced sensorimotor dysfunction. The experiment assessed both stride length (distance cut in centimetres by one foot through the gait cycle using 3 hind paw strides when the animal moves at constant pace; [23]) and stride width (mean of side-to-side distance between the two hind paws in three consecutive strides; [24]). At the beginning of experiment and before injecting 3-NP, rats were trained to cross directly into their home *via* a horizontal path; afterwards the hind limbs were immersed in a non-toxic paint (Crayola LLC, NY, USA), and the animals were allowed to travel the corridor to their home cage on a piece of paper.

#### Pole test

Muscle rigidity and postural instability were assessed by this test, by placing each rat, head-up, on top of a wooden pole (69 cm long, 8–10 cm in diameter) that was placed in the home cage. The time required for each animal to descend down the pole to the ground was determined and the mean time of 5 trials for each rat was calculated [25].

#### Cylinder test

Rats were placed in a clear plexiglass cylinder (height/diameter = 30/20 cm) and video recorded for 5 min. When the animal raises forelimbs above shoulder level and touches the cylinder wall with either one or both forelimbs, this is considered as a rear. Additionally, rat must remove both forelimbs from the cylinder wall and touch the cylinder base before another rear was scored. Hence, total rearing time, in addition to rearing frequency in 5 minutes were determined [26,27].

### Striatal processing

After behavioural testing, all animals were sacrificed by decapitation under thiopental sodium (5 mg/kg) anaesthesia [28]. Brains were rapidly dissected out and the striata were isolated and stored at -80°C. The right striata of 8 animals were homogenized in radioimmunoprecipitation assay (RIPA) buffer (50 mM Tris HCl, pH 8, 150 mM NaCl, 1% Triton X-100, 0.5% sodium deoxycholate and 0.1% SDS) provided with protease inhibitor cocktail and used for the estimation of phosphorylated cAMP response element binding protein (*p-*S133 CREB), *p-*S473 Akt, and toll like receptor (TLR)-4. The left striata of the previous 8 rats were homogenized in ice cold phosphate buffer saline (PBS; pH = 7.4) and used for the ELISA determination of interleukin (IL)-6, IL-10, nuclear factor kappa B **(**NF-κB) p65, phosphorylated glycogen synthase kinase-3 β (*p*-S9 GSK-3β), Janus Kinase 2 (*p*-Y1007/1008 JAK-2), signal transducers and activators of transcription (*p-*Y705 STAT-3) and suppressor of cytokine signalling 3 (SOCS3) in addition to, caspase-3 activity. These parameters were normalized to protein content, measured according to **Bradford** [29]. The striata of the remaining 4 rats were used to determine the immunohistochemical protein expression of tyrosine hydroxylase (TH) and caspase-3, besides the histopathological assessment.

#### Quantification of striatal NF-κB p65, IL-6, IL-10, *pY1007/1008* JAK-2, *p*Y705 STAT3, SOCS3, *and p*S9 GSK-3β by ELISA technique

The following parameters were assessed using the corresponding ELISA kit with the source and catalogue number mentioned in parenthesis. Striatal NF-κB p65 (MyBioSource; CA, USA; cat# MBS261874) IL-6 (RayBiotech; Georgia, USA; cat# ELR-IL6); IL-10 (R&D; Minneapolis, USA; cat# R1000); *p-*Y1007/1008 JAK-2 (Invitrogen; CA, USA; cat# KHO5621); *p-*Y705 STAT-3 (Creative Diagnostics; NY, USA; cat# DEIA4233); and SOCS3 (MyBiosource; CA, USA; cat# MBS2515983); *p-*S9 GSK-3β (LifeSpan BioSciences, Inc.; Seattle, WA, USA; cat# LS-F1521). All the procedures were performed according to the manufacturers’ instructions.

#### Assessment of *p-*S133 CREB, *p-*S473 Akt, and TLR4 by Western Blot technique

Following striatal protein quantification (Bio-Rad Protein Assay Kit, CA, USA), 10 μg proteins of each sample were separated by SDS polyacrylamide gel electrophoresis and transferred to a nitrocellulose membrane using a semi-dry transfer apparatus (Bio-Rad, CA, USA). Membranes were then soaked in 5% non-fat dry milk to block non-specific binding sites. Afterward, the membrane was incubated with anti- *p-*S133 CREB (1:250; cat#: PA1-851B), anti- *p*S473 Akt (1:100; cat#: OMA1-03061) and anti-TLR4 (1:100; cat#: MA5-16216) polyclonal antibody (ThermoFisher Scientific, MA, USA) overnight at 4°C on a roller shaker. Next, membranes were probed with horseradish peroxidase-conjugated goat anti-rat immunoglobulin (Dianova, Hamburg, Germany). Finally, the blots were developed with enhanced chemiluminescence detection reagent (Amersham Biosciences, IL, USA). Protein was quantified by densitometric analysis using a scanning laser densitometer (GS-800 system, Bio-Rad, CA, USA). Results were expressed as arbitrary units (AU) after normalization for β-actin protein expression.

#### Immunohistochemical assay of tyrosine hydroxylase (TH) and caspase-3

For immunohistochemical examination, striatal tissues were fixed in 10% neutral buffered formalin overnight and then embedded in paraffin, to be sectioned at 4-μm thickness. The paraffin embedded sections were deparaffinized in xylene, and hydrated by ethyl alcohol in decreasing concentrations (100%, 95% and 70%). Antigen retrieval was achieved by heating slides (in a 200 ml Coplin jar filled with 10 mM citrate buffer) in a commercial microwave oven operating at a frequency of 2.45 GHz and 600 W power setting. After two heating cycles of 5 minutes each, slides were allowed to cool at room temperature and thoroughly washed in PBS (pH 7.4). Striatal sections were stained by applying the labelled streptavidin–biotin–peroxidase method according to the manufacturer staining protocol (Vectastain Elit ABC peroxidase kit, Vector Laboratories, Burlingame, CA, USA). Brifly, endogenous peroxidase activity was quenched by incubating the specimen for 5 min with 3% hydrogen peroxide. Sections were incubated with corresponding primary antibody for TH (Biorbyt Co., Cambridge, UK; cat#:orb43362) and caspase-3 (Abcam Co., Cambridge, UK;cat#:ab184787) at 4°C overnight. After conjugation with streptavidin–biotin–peroxidase complex, colouring was performed with 3, 30-diaminobenzidine substrate-chromogen and hematoxylin was used for counter staining the reacted caspase-3 (1:100) and TH (1:100) antibodies. Microscopical examination was performed and five serial fields were captured at 400× magnification and the intensity of reaction into striatal area for each antibody among different groups was determined using Leica Application Suite imaging software (Leica Microsystems, Germany).

#### Determination of caspase-3 activity

Caspase-3 activity was estimated using a colorimetric assay kit (R&D Systems, Inc., USA; cat#: BF3100). The results were expressed as fold increase in optical density relative to the normal group.

### Histopathological analysis

The paraffin embedded slides were stained using H&E stain for histopathological evaluation. Neuronal cells stained with H&E were viewed and the striatal neurons were outlined, then the pathological changes in striatum were examined at high power (×400 magnification) in each group. In H & E staining, each section was assigned a damage score between 0 and 3 for each of five parameters, namely, necrosis of neurons, neurophagia, cellular edema, congestion of blood vessels and focal gliosis. The scores for the five parameters measured for each rat were summed to obtain the “total histology score”, being maximally 15 (three being the maximum for the five parameters examined). An experienced pathologist who was blinded to the experiment groups performed all histopathological examinations.

### Statistical analysis

Data are expressed as means ± SEM. GraphPad Prism^®^ software package, version 6 (GraphPad Software Inc., CA, USA) was used to carry out all statistical tests. For parametric analysis, multiple comparisons were performed using one way analysis of variance (ANOVA) test followed by Tukey's Multiple Comparison test. For non-parametric data, one-way analysis of variance test (Kruskel-Wallis Test) followed by Dunn’s multiple comparisons test were used. P<0.05 was set as the significance limit for all comparison.

## Results

Groups treated with cilostazol alone without 3-NP injections showed no significant difference as compared to the normal control group, thus, all comparisons were carried against the normal control group.

### Cilostazol ameliorated 3-NP-induced neurobehavioral derangements

**Fig 1:**
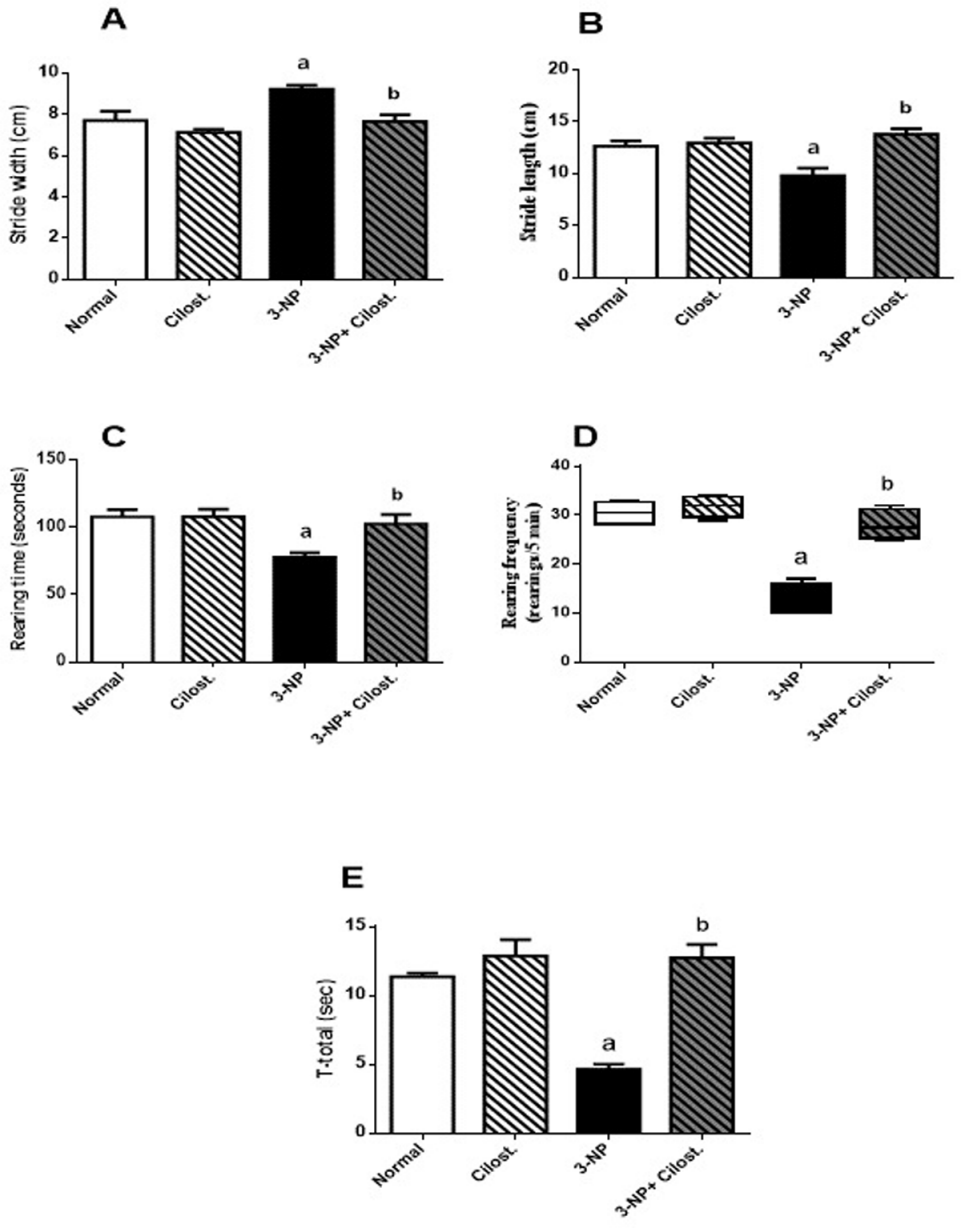
**Effect of cilostazol (100 mg/kg, p.o) on (A) stride width, (B) stride length, (C) rearing time, (D) rearing frequency, and (E) T−total in 3-NP-treated rats.** Non-parametric data are presented as median (max-min) using Kruskel–Wallis test followed by Dunn’s as a *post-hoc* test. Parametric data are presented as mean ± SEM. Statistical analysis was performed using one-way ANOVA followed by Tukey's Multiple Comparison. ^a^ Significantly different from normal group at P<0.05, ^b^ Significantly different from 3-NP- treated group at P<0.05.

**Fig 1** highlights the 3-NP-induced behavioural abnormalities. Using the manual gait test, 3-NP injection suppressed locomotion and motor coordination evidenced by the (A) increased stride width along with a shortening of the (B) stride length. However, concomitant administration of cilostazol improved muscle coordination and hindered the 3-NP effect significantly. Additionally, using the cylindrical test 3-NP-lesioned animals exhibited a progressive decrease in the (C) rearing time and (D) rearing frequency (rearing/5 min) in comparison to normal control group. Treatment with cilostazol, on the other hand, increased both the number and time of rearing significantly compared to 3-NP group. Finally, results of the pole test revealed that insulted rats showed a significant decrease in (E) T−total compared to normal group, whereas cilostazol reverted the 3-NP effect.

### Cilostazol alleviated 3-NP-induced alternations in tyrosine hydroxylase (TH) and caspase-3

**Fig 2:**
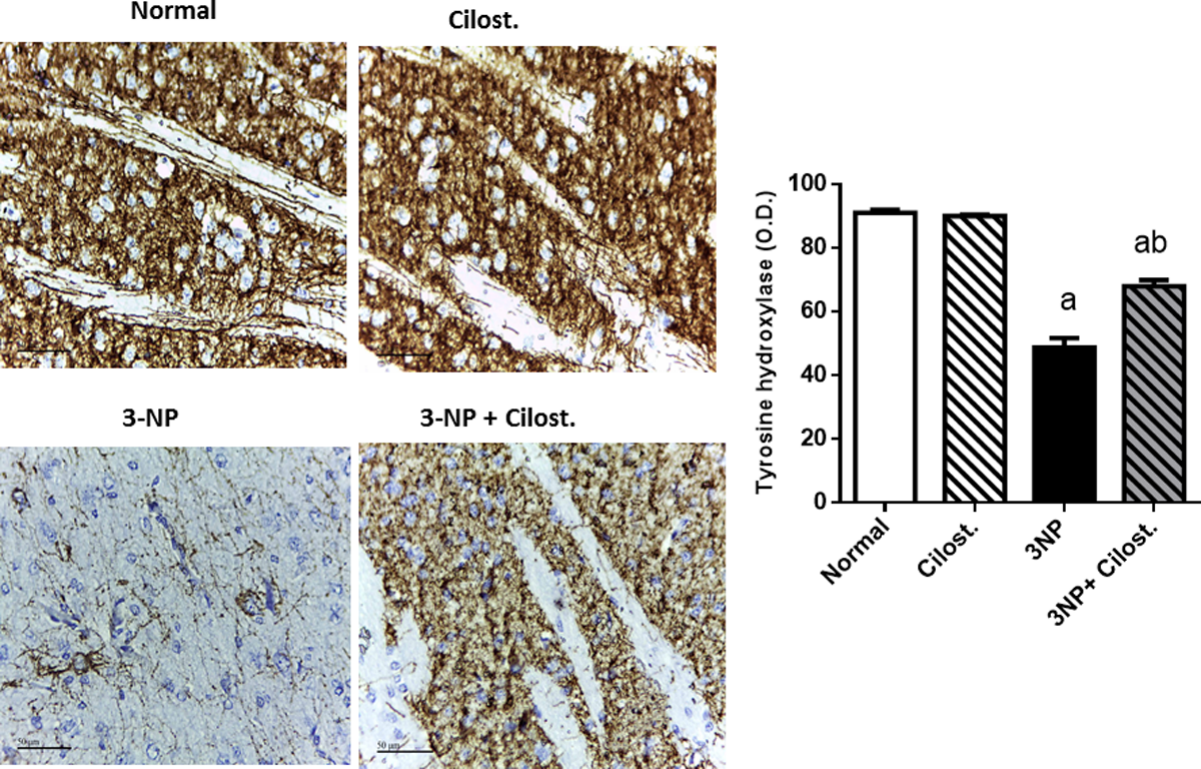
Effect of cilostazol (100 mg/kg, p.o) on protein expression of TH in 3-NP-treated rats. Data are presented as mean ± SEM. Statistical analysis was performed using one-way ANOVA followed by Tukey's Multiple Comparison. ^a^ Significantly different from normal group at P<0.05, ^b^ Significantly different from 3-NP- treated group at P<0.05, ^ab^ Significantly different from both normal and 3-NP- treated groups at P<0.05.

**Fig 3:**
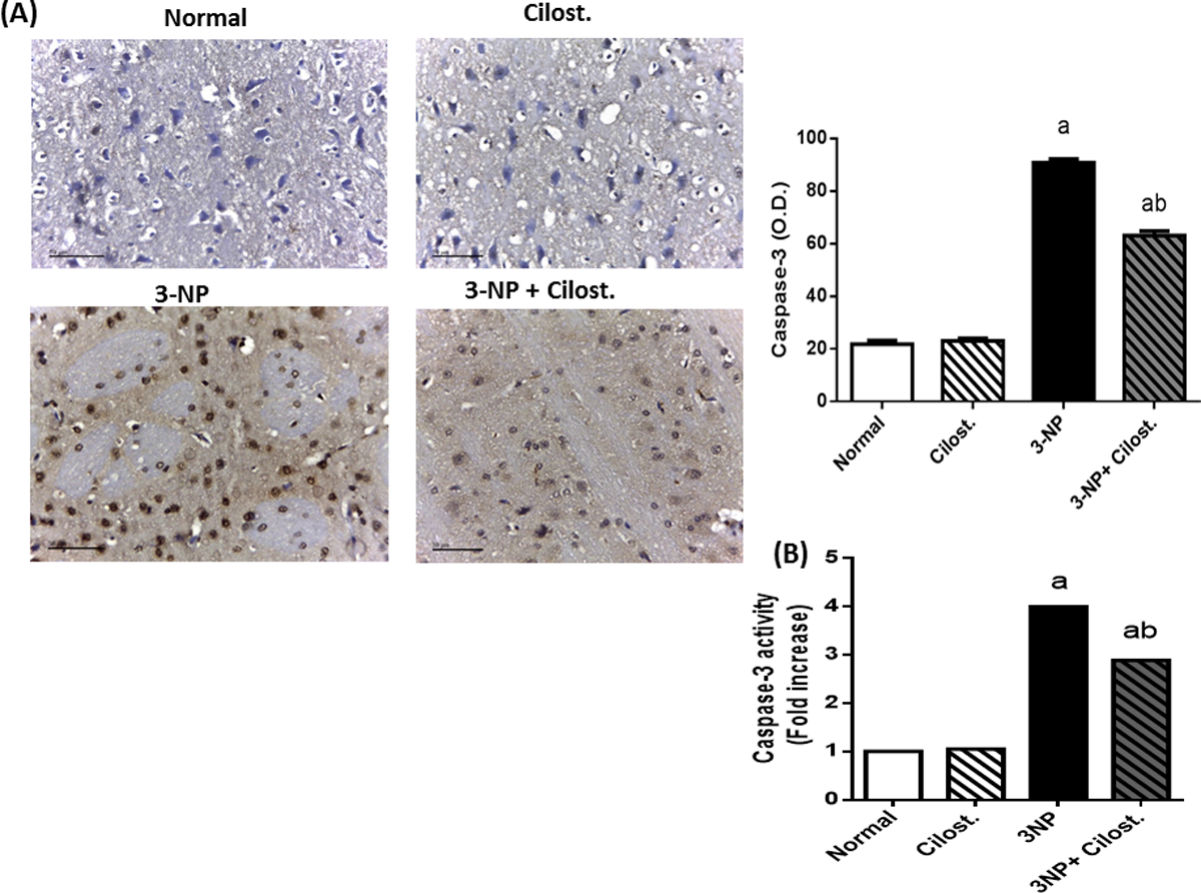
**Effect of cilostazol (100 mg/kg, p.o) on (A) protein expression and (B) activity of caspase-3 in 3-NP-treated rats.** Caspase-3 protein expression is presented as mean ± SEM and caspase-3 activity is presented as fold increase relative to normal group. Statistical analysis was performed using one-way ANOVA followed by Tukey's Multiple Comparison. ^a^ Significantly different from normal group at P<0.05, ^b^ Significantly different from 3-NP- treated group at P<0.05, ^ab^ Significantly different from both normal and 3-NP- treated groups at P<0.05.

The deficit motor performance observed after 3-NP treatment was associated with a reduction in the optical density of striatal TH antibody immuno-reactivity as compared to normal group (**Fig 2**). Simultaneously, 3-NP-insulted rats exhibited an upsurge in caspase-3 expression as shown by immunohistochemical assay as well as caspase-3 activity to reach 4 fold the normal group (**Fig 3**).

Nevertheless, co-administration of cilostazol protected striatal neuronal cells from degenerative apoptotic death as it suppressed both the expression and the activity of pro-apoptotic protein caspase-3 and consequently preserved dopaminergic neurons as shown by increased immunostaining density of striatal TH as compared to 3-NP control group.

### Cilostazol modulated the striatal contents of IL-6, IL-10, NF-κB p65 and TLR-4

**Fig 4:**
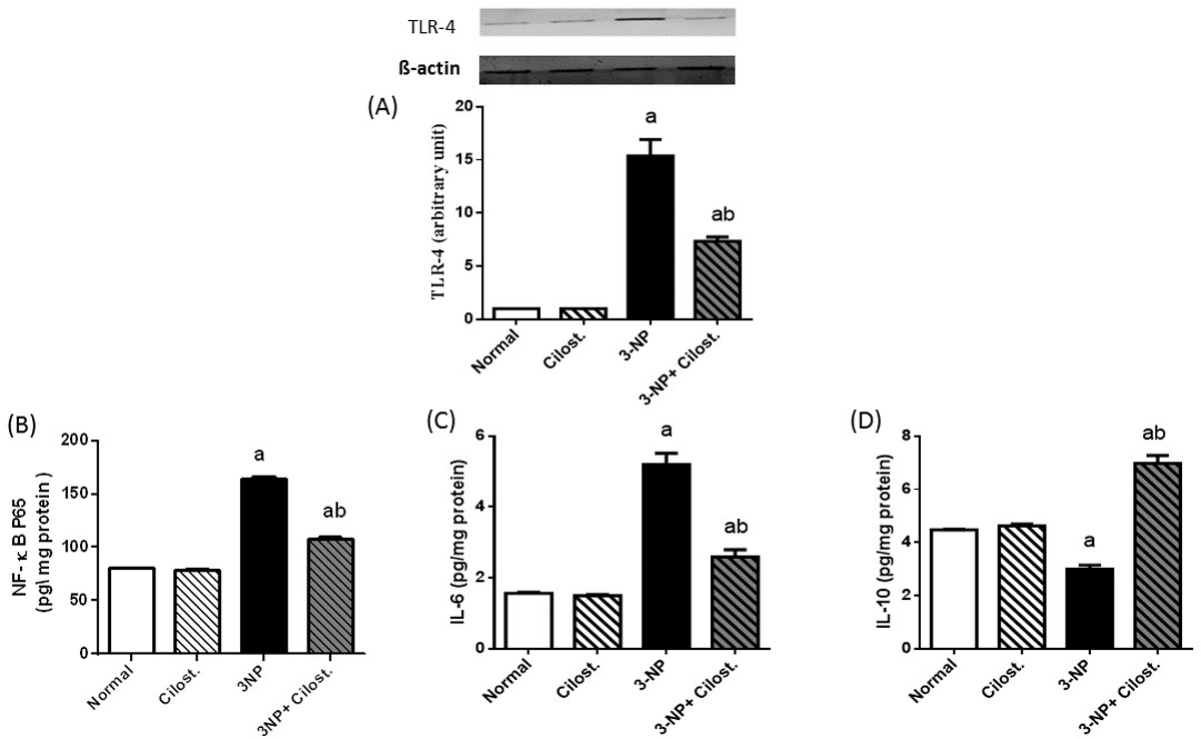
**Effect of cilostazol (100 mg/kg, p.o) on striatal protein expression/contents of (A) TLR-4, (B) NF-κB p65, (C) IL-6, and (D) IL-10 in 3-NP-treated rats.** Data are presented as mean ± SEM. Statistical analysis was performed using one-way ANOVA followed by Tukey's Multiple Comparison. ^a^ Significantly different from normal group at P<0.05, ^b^ Significantly different from 3-NP- treated group at P<0.05, ^ab^ Significantly different from both normal and 3-NP- treated groups at P<0.05.

Untreated rats with 3-NP insult (**Fig 4**) showed a marked elevation in (A) TLR-4, (B) NF-κB p65 and (C) IL-6, along with a significant fall in the (D) IL-10 content. Conversely, presence of cilostazol impeded the 3-NP action, as it heightened the anti-inflammatory cytokine IL-10, while reduced NF-κB p65, IL-6 and TLR-4.

### Cilostazol enhanced the striatal p-(S473) Akt, p-(S9) GSK-3β, and p-(S133) CREB

**Fig 5:**
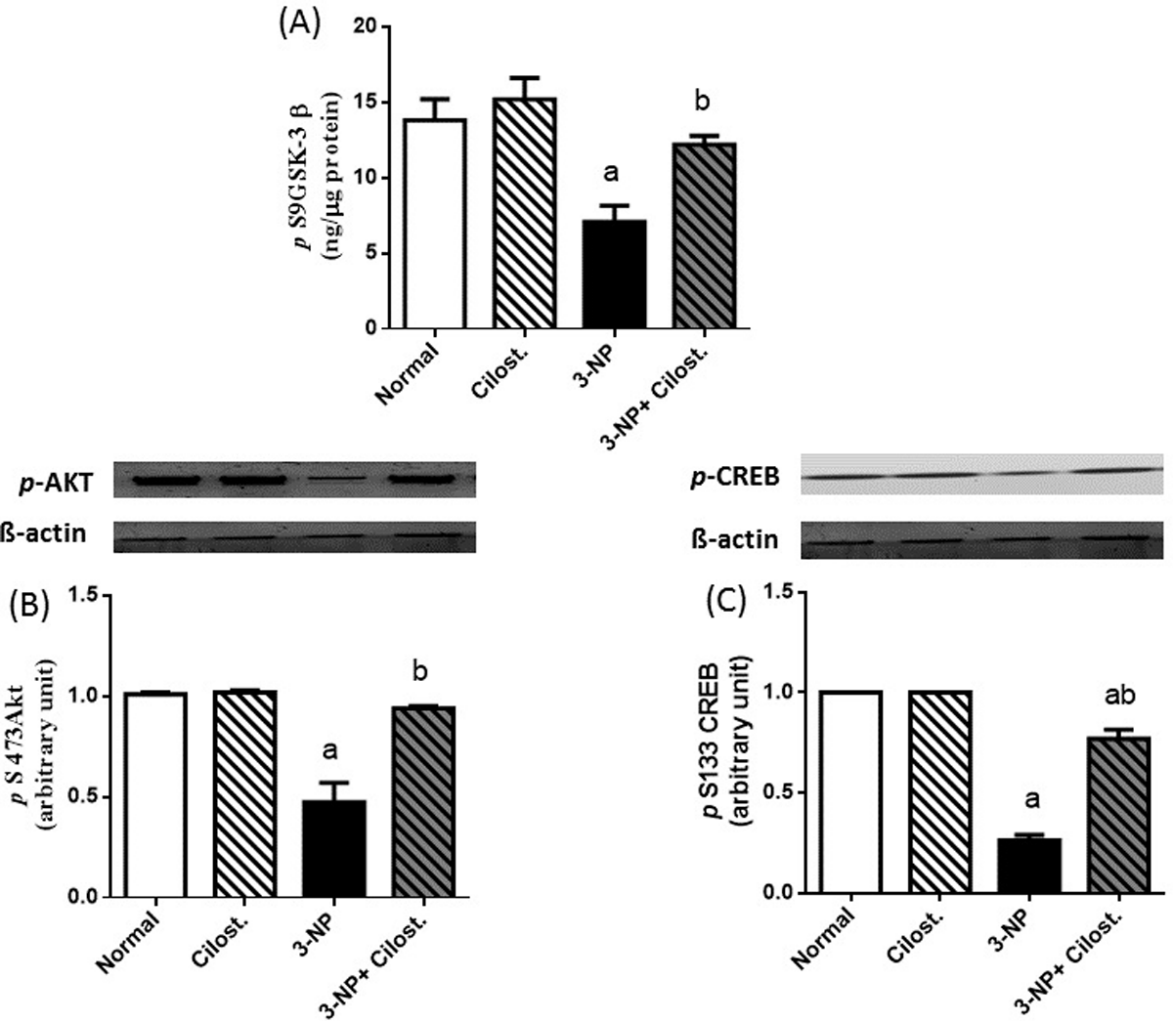
**Effect of cilostazol (100 mg/kg, p.o) on striatal protein expression/contents of (A) *p*-GSK-3β, (B) *p*-Akt, and (C) *p*-CREB in 3-NP-treated rats.** Data are presented as mean ± SEM. Statistical analysis was performed using one-way ANOVA followed by Tukey's Multiple Comparison. ^a^ Significantly different from normal group at P<0.05, ^b^ Significantly different from 3-NP- treated group at P<0.05, ^ab^ Significantly different from both normal and 3-NP- treated groups at P<0.05.

As depicted in **Fig 5**, 3-NP abated the striatal (A) *p*-GSK-3β, as well as the protein expression of (B) *p***-**Akt and (C) *p***-**CREB, as compared to normal control group. On the other hand, cilostazol increased the phosphorylation of the three signalling molecules, as compared to 3-NP-lesioned rats.

### Cilostazol modulated the striatal contents of p-(Y1007/1008) JAK-2, p-(Y705) STAT3, and SOCS3

**Fig 6:**
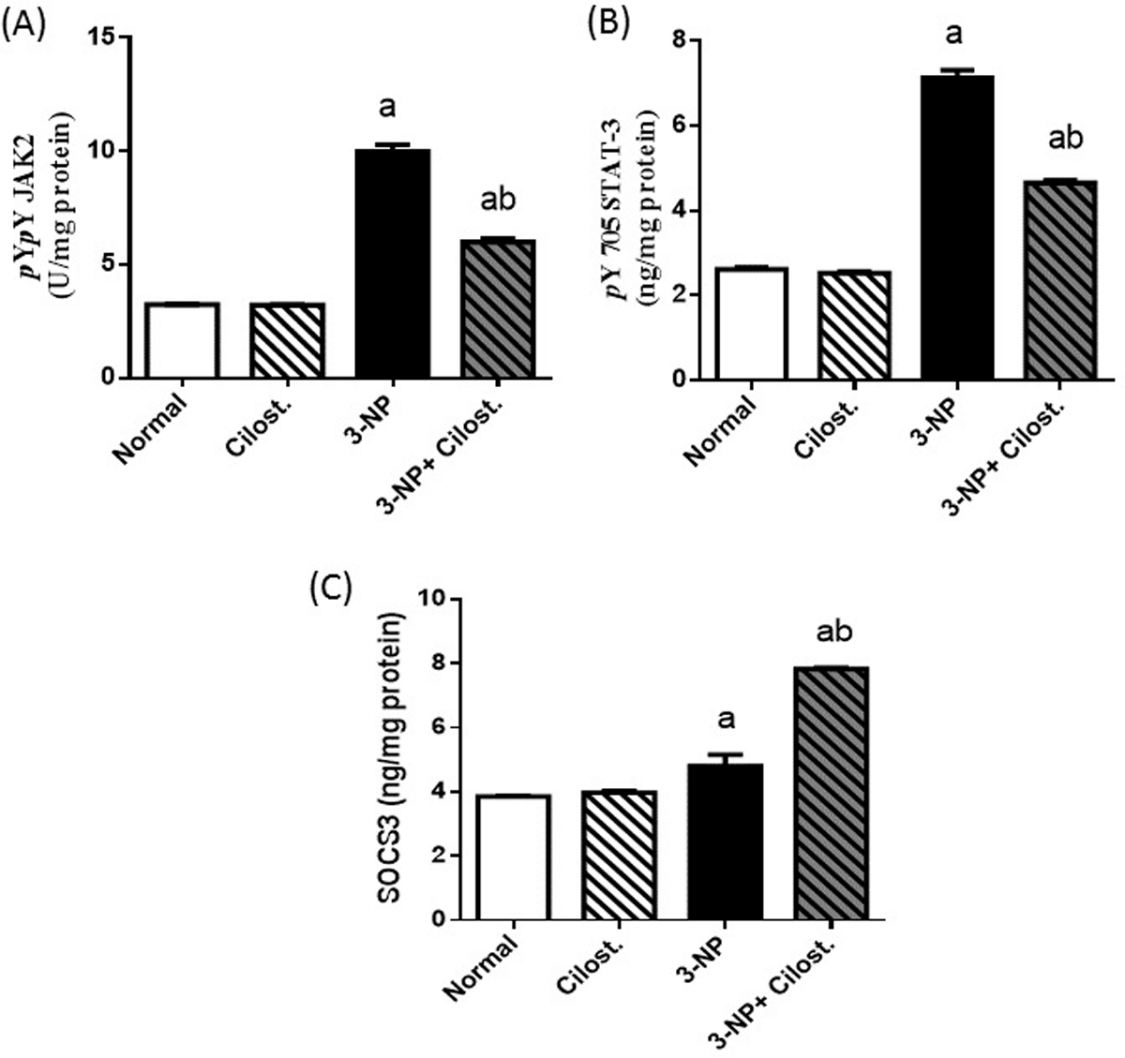
**Effect of cilostazol (100 mg/kg, p.o) on striatal contents of (A) *p*-JAK-2, (B) *p*-STAT-3, and (C) SOCS3 in 3-NP treated rats.** Data are presented as mean ± SEM. Statistical analysis was performed using one-way ANOVA followed by Tukey's Multiple Comparison. ^a^ Significantly different from normal group at P<0.05, ^b^ Significantly different from 3-NP- treated group at P<0.05, ^ab^ Significantly different from both normal and 3-NP- treated groups at P<0.05.

**Fig 6**demonstrated that 3-NP boosted striatal (A) *p*-JAK-2 and its downstream (B) *p*-STAT3, effects that were paralleled by an increase in (C) SOCS3 content, as compared to normal control group. Contrariwise, the coadministration of cilostazol suppressed both *p*-JAK-2 and *p*-STAT3, but caused a further rise in SOCS3 content, as compared to 3-NP.

### Cilostazol-preserved neurons in striata against 3-NP-induced degeneration

**Fig 7:**
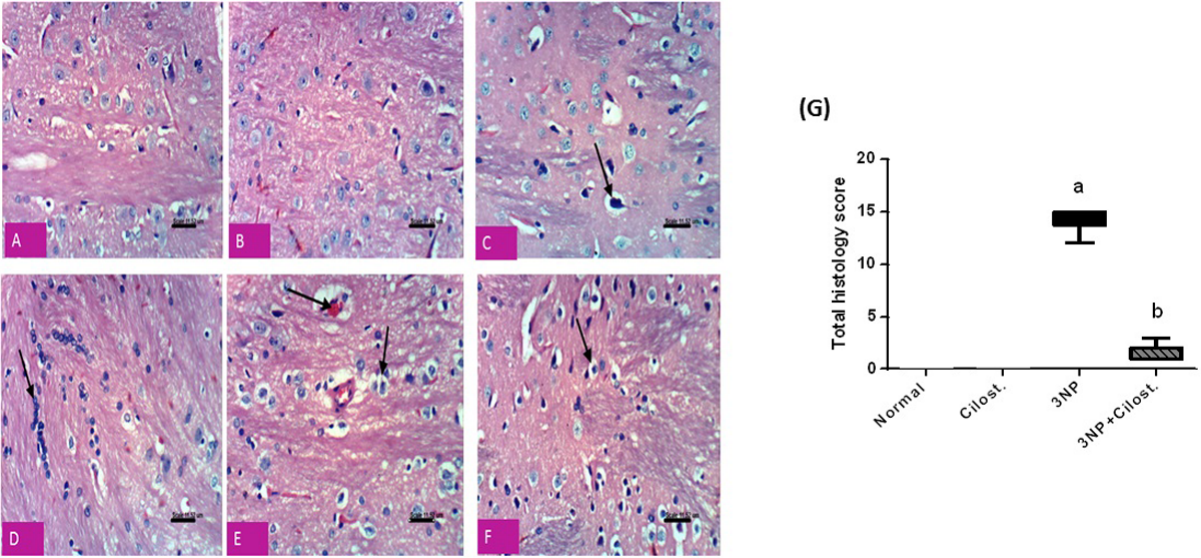
Effect of cilostazol (100 mg/kg, p.o) on striatal histopathological photomicrographs in 3-NP-treated rats. Sections A and B show normal neuronal structure of rats receiving saline and cilostazol, respectively. Section of (C) 3-NP treated group show a significant neuronal damage with severe neuronal necrosis and neuronophagia, (D) focal gliosis, and (E) cellular edema with congested blood capillaries. However, section of (F) cilostazol treated group show an improvement in histopathological changes induced by 3-NP, except for mild cellular edema. (G) Total histology score, data are expressed as box plots of the median of 6 animals. Statistical analysis was done using Kruskal-Wallis test followed by Dunn's test. ^a^ Significantly different from normal group at P<0.05, ^b^ Significantly different from 3-NP- treated group at P<0.05.

Histopathological photomicrographs (**Fig 7**) show that (C) 3-NP section caused a significant neuronal damage compared to sections of the normal (A) saline and (B) cilostazol groups. 3-NP resulted in (C) severe neuronal necrosis and neuronophagia, (D) focal gliosis, and (E) cellular edema with congested blood capillaries. The total histology score was markedly increased in rats with 3-NP insults (G). On the other hand, co-administration of cilostazol resulted in a prominent decrease in total histology score (G) as well as improvement in histopathological changes induced by 3-NP, except for mild cellular edema (F).

## Discussion

The present study highlighted the potential neuroprotective effect of cilostazol, the phosphodiesterase III inhibitor, in a 3-NP-induced HD model. Cilostazol strongly ameliorated 3-NP-induced neuro-inflammation and striatal degeneration. It amended the 3-NP-induced lesions in the striatal dopaminergic neurons as evidenced by the increased protein expression of TH along with the marked improvement in histopathological changes. These restorations were reflected on the functional tests, where cilostazol enhanced neurobehavioral performance (locomotor activity, motor coordination, muscle rigidity, and postural stability) as well. The present study shed light on some of the multiple downstream signalling targets of cyclic nucleotide that may account for the cilostazol neuroprotective effects.

Cilostazol signified its anti-inflammatory effect by suppressing the inflammatory event resulting from the 3-NP-induced elevation in NF-κB p65, IL-6, and TLR-4 protein expression. Indeed, the inflammatory cascades provoked as a response to many types of brain insults are pivotal to control and counteract detrimental effects on neurons. However, severe or chronic inflammation, can itself damage neurons due to the excessive activation of astrocytes and microglia via the utilization of TLRs [30,31]. These receptors recognize damage-associated molecular patterns and mediate host-inflammatory responses to injury through the activation of transcription factors, such as NF-κB p65, as shown herein, with the subsequent production of pro-inflammatory cytokines, as IL-6, TNF-α and IL-1β [32,33].

The upregulation of TLR-4 expression has been reported in Parkinson’s disease and multiple systems atrophy post-mortem brain tissue, suggesting clinical relevance of TLR-4 in the progression of many widespread neurodegenerative diseases [34,35]. Moreover, chronic or excessive activation of glia by IL-6 is a common denominator in neuronal loss in several neurodegenerative diseases [36]. In the present study, injection of 3-NP markedly increased the striatal IL-6, which in turn increases TLR-4, these results concur with that of Tamandl et al. [37] who showed that IL-6 upregulated TLR-4 resulting in hyper-responsiveness to lipopolysaccharides (LPS). The anti-inflammatory effect of cilostazol can be attributed partly to the inhibition of the key inflammatory transcription factor NF-κB p65 that entails its downstream cytokines as reported herein and previously [38]. In support to the current findings, Park et al. [39] reported that cilostazol was able to suppress the expression of TLR-4 along with cytokine production in macrophages from patients with rheumatoid arthritis.

Apart from abating the inflammatory mediators, cilostazol spared the anti-inflammatory cytokine IL-10 possibly by the phosphorylation/inactivation of GSK-3β. In fact, GSK3β, a crucial modulator of innate inflammatory processes, potently suppresses the production of the cytokine IL-10, while concurrently augments the production of pro-inflammatory cytokines in response to TLR-4 signalling pathway [40,41]. Additionally, cilostazol has activated/ phosphorylated protein kinase B (PKB or Akt) and the downstream molecule CREB besides GSK-3 β to pin down its anti-inflammatory character and to concur with previous studies in different models [42,43]. Ample evidence have emphasized the neuroprotective effect of *p-*Akt/*p-*GSK-3β in different models of neurotoxicity in rats [44–46]. Therefore, the ability of 3-NP to reduce the phosphorylated forms of the Akt/GSK-3β/CREB cue certified the occurrence of inflammatory milieu to highlight the role of neuro-inflammation in the progression of 3-NP-induced striatal degeneration, as confirmed herein and reported earlier [6,47]. Cilostazol-mediated activation of Akt is responsible for the current inactivation/ phosphorylation of GSK-3β at the serine site to match previous work [48] and to endorse the activation of many downstream transcription factors, such as CREB [49]. This can provide a molecular understanding of how GSK-3β inactivation promotes an anti-inflammatory immune response after TLR activation. Inactive/phosphorylated GSK-3β enhances CREB association with CREB binding protein (CBP), while reducing the interaction between NF-κB p65 with CBP required for its optimal transcriptional activity [40]. A previous *in vitro* study[50] harmonizes with the present findings, where cilostazol significantly increased the expression of *p-*CREB and decreased nuclear NF-κB p65 (level and DNA binding activity). Moreover, *p-*CREB plays an essential role in the production of IL-10 via binding to its promotor region to induce its transcription [51] to be one explanation for the boosted IL-10 content.

Besides its anti-inflammatory role, *p*-CREB plays a role in the improved motor activity noticed herein via increasing the gene transcription of TH as stated previously by Park et al. [50] and as reported in our findings. In the present work, cilostazol-mediated activation of CREB was associated also by an increase in the protein expression of TH to increase the production of dopamine and to support the intact functional dopaminergic neurons [52].

Apart from its role in inflammation, the Akt/GSK-3β/CREB axis plays also a part in cell survival via regulating apoptosis, a cellular death type that has been involved in various neurodegenerative disorders including HD [53,54]. Activation of one or more TLRs in neurons and glial cells increases the vulnerability of neurons to apoptosis [55]. In parallel, downregulation of the defence signal Akt/GSK-3β promotes the neuronal death partially via suppressing CREB activity that correlates with the expression of many survival and proliferation genes [56]. The current immuno-histological examination along with the enzyme activity test revealed that cilostazol markedly curbed caspase-3 activity that was enhanced in 3-NP treated rats, to emphasize its neuroprotective potential. This effect is possibly linked to its modulatory effect on TLR-4 expression and *p-*Akt/*p-*GSK-3β/*p-*CREB signalling pathway. In line with these findings, earlier studies divulged that cilostazol possesses an anti-apoptotic effect via enhancing the phosphorylation/activation of CREB and increasing the anti-apoptotic Bcl-2 in cerebral ischemia model [17,43] and in the white matter region after cerebral hypoperfusion [18].

In our study, the second molecular signalling pathway that was modulated by cilostazol is the *p*-JAK-2/*p*-STAT-3/SOCS3 pathway to nail down its anti-inflammatory effect. Activation of *p-*JAK-2/*p-*STAT-3 pathway plays a dire role in neuro-inflammatory diseases [57,58]**,** where upon binding to its receptor, IL-6 triggers the phosphorylation of JAK-2 with the subsequent phosphorylation of its downstream molecule STAT-3. Dimerized *p*-STAT-3 molecules translocate into the nucleus to augment the transcription factor NF-κB [59], a fact that adds to the increased content of this transcription factor. Additionally, nuclear STAT-3 by binding to DNA increases cytokine gene expression, suggesting that by activating STAT-3, IL-6 promotes its own production in a feed-forward loop leading to sustained inflammation [60]; these events support our results. Such inflammatory cascade induces SOCS3 to act as a negative feedback regulator to inhibit inflammation running out of control [61]. Beurel and Jope. [62] previously reported that during neuro-inflammation the glial production of IL-6 prerequisites a strong integration between *p-*STAT-3 and active GSK-3β, where, GSK-3β was found to be crucial for the activation of STAT3 in primary astrocytes by promoting its tyrosine phosphorylation (Y 705)[63]. Hence, the interference with either the activity of STAT-3 or GSK-3β was associated with a marked suppression in the release of IL-6 to confirm the key role of both molecules in promoting IL-6 production by glial cells [62].

In agreement with the previous *in vitro* study of Park et al. [50], cilostazol herein impeded the 3-NP-associated elevation in IL-6/*p-*JAK-2/*p-*STAT-3 to accentuate the neuroprotective role mediated by suppressing this cue. These data synchronize with that of Qin et al. [64], who also underlined the beneficial effects of inhibiting JAK/STAT pathway in α-synuclein-induced neuroinflammation and dopaminergic neurodegeneration. In what appears to be a vicious cycle, STAT-3 also induces the expression of TLR-4 with proposed binding sites of STAT-3 in the TLR4 promoter region [65,66]. This could partially explain the marked increase in both STAT-3 content and TLR-4 expression after 3-NP administration and their co-inhibition in cilostazol treated rats.

The ability of cilostazol to dampen *p-*STAT-3 could be attributed to the inactivation of GSK-3β, since GSK-3β profoundly activates STAT-3 [63]. As a compensatory response against the inflammatory events, SOCS-3 was elevated in the 3-NP rats; however, this rise was not enough to oppose the inflammatory upsurge in the 3-NP group. Nevertheless, concomitant administration of cilostazol increased further the production of SOCS-3 to play its role in quelling the inflammatory flare and to support the findings of Gaudy et al. [67], who proved that increased cAMP induces SOCS3 to negatively regulate JAK/STAT signalling pathway. Intriguingly, SOCS3 has a regulatory role in the function of STAT3 induced by IL-6, but not that of IL-10 due to a different affinity of SOCS3 to the two receptors [68]. In a mutual intervention, the cilostazol-induced SOCS-3 expression could indorse the production of IL-10 [69], which in turn, can induce further the release of SOCS3 [70, 71]. In addition, the ability of cilostazol to trigger PI3K/Akt/*p*-GSK-3β trajectory may participate in increasing IL-10 production [72].Thus, the anti-inflammatory effect of IL-10 mediated through STAT-3 is preserved along with that of PI3K/Akt/GSK-3β pathway, which implicated in both the production and responses to IL-10 [72].

In conclusion, the present study highlighted the neuroprotective potential of cilostazol against 3-NP-induced HD model via modulating the crosstalk between TLR-4, Akt/GSK-3β/CREB and JAK-2/STAT3/SOCS-3 signalling pathways to provide a promising therapeutic tool slowing the progression of HD.

## References

[1] G. Vonsattel JP, DiFiglia M. Huntington Disease. J Neuropathol Exp Neurol. 1998; 57: 369–384. 10.1097/00005072-199805000-00001 9596408

[2] Leegwater-Kim J, Cha J-HJ. The Paradigm of Huntington’s Disease: Therapeutic Opportunities in Neurodegeneration. NeuroRx. 2004; 1: 128–138. Available: http://www.ncbi.nlm.nih.gov/pmc/articles/PMC534918/%5Cnhttp://www.ncbi.nlm.nih.gov/pmc/articles/PMC534918/pdf/neurorx001000128.pdf 10.1602/neurorx.1.1.128 15717013 PMC534918

[3] Túnez I, Tasset I, Pérez-De La Cruz V, Santamaría A. 3-Nitropropionic acid as a tool to study the mechanisms involved in Huntington’s disease: past, present and future. Molecules. 2010; 15: 878–916. 10.3390/molecules15020878 20335954 PMC6263191

[4] Hanna DMF, Tadros MG, Khalifa AE. ADIOL protects against 3-NP-induced neurotoxicity in rats: Possible impact of its anti-oxidant, anti-inflammatory and anti-apoptotic actions. Prog Neuro-Psychopharmacology Biol Psychiatry. 2015; 60: 36–51. 10.1016/j.pnpbp.2015.02.005 25689821

[5] Alexi T, Hughes PE, Faull RL, Williams CE. 3-Nitropropionic acid’s lethal triplet: cooperative pathways of neurodegeneration. Neuroreport. 1998; 9: R57–R64. 9721909 10.1097/00001756-199808030-00001

[6] Rodriguez AI, Willing AE, Saporta S, Cameron DF, Sanberg PR. Effects of Sertoli cell transplants in a 3-nitropropionic acid model of early Huntington’s disease: A preliminary study. Neurotox Res. 2003; 5: 443–450. 10.1007/BF03033174 14715448

[7] Lu P. Combinatorial Therapy with Neurotrophins and cAMP Promotes Axonal Regeneration beyond Sites of Spinal Cord Injury. J Neurosci. 2004; 24: 6402–6409. 10.1523/JNEUROSCI.1492-04.2004 15254096 PMC6729552

[8] Atkins CM, Oliva AA, Alonso OF, Pearse DD, Bramlett HM, Dietrich WD. Modulation of the cAMP signaling pathway after traumatic brain injury. Exp Neurol. 2007; 208: 145–158. 10.1016/j.expneurol.2007.08.011 17916353 PMC2141537

[9] Knott EP, Assi M, Rao SNR, Ghosh M, Pearse DD. Phosphodiesterase inhibitors as a therapeutic approach to neuroprotection and repair. Int J Mol Sci. 2017; 18 10.3390/ijms18040696 28338622 PMC5412282

[10] Bollen E, Prickaerts J. Phosphodiesterases in neurodegenerative disorders. IUBMB Life. 2012; 64:965–970. 10.1002/iub.1104 23129425

[11] Conti M, Beavo J. Biochemistry and Physiology of Cyclic Nucleotide Phosphodiesterases: Essential Components in Cyclic Nucleotide Signaling. Annu Rev Biochem. 2007; 76: 481–511. 10.1146/annurev.biochem.76.060305.150444 17376027

[12] Kimura Y, Tani T, Kanbe T, Watanabe K. Effect of cilostazol on platelet aggregation and experimental thrombosis. Arzneimittelforschung. 1985; 35:1144–1149. Available: http://www.ncbi.nlm.nih.gov/pubmed/4074426 4074426

[13] Gotoh F, Tohgi H, Hirai S, Terashi A, Fukuuchi Y, Otomo E, et al Cilostazol stroke prevention study: A placebo-controlled double-blind trial for secondary prevention of cerebral infarction. J Stroke Cerebrovasc Dis. 2000; 9: 147–157. 10.1053/jscd.2000.7216 24192020

[14] Choi JM, Shin HK, Kim KY, Lee JH, Hong KW. Neuroprotective effect of cilostazol against focal cerebral ischemia via antiapoptotic action in rats. J Pharmacol Exp Ther. 2002; 300: 787–793. 10.1124/jpet.300.3.787 11861782

[15] Lee JH, Lee Y-K, Ishikawa M, Koga K, Fukunaga M, Miyakoda G, et al Cilostazol reduces brain lesion induced by focal cerebral ischemia in rats—an MRI study. Brain Res. 2003; 994: 91–8. 10.1016/j.brainres.2003.09.021 14642452

[16] Honda F, Imai H, Ishikawa M, Kubota C, Shimizu T, Fukunaga M, et al Cilostazol attenuates gray and white matter damage in a rodent model of focal cerebral ischemia. Stroke. 2006; 37: 223–8. 10.1161/01.STR.0000196977.76702.6d 16339464

[17] Lee JH, Park SY, Shin YW, Hong KW, Kim CD, Sung SM, et al Neuroprotection by cilostazol, a phosphodiesterase type 3 inhibitor, against apoptotic white matter changes in rat after chronic cerebral hypoperfusion. Brain Res. 2006; 1082: 182–191. 10.1016/j.brainres.2006.01.088 16516167

[18] Watanabe T, Zhang N, Liu M, Tanaka R, Mizuno Y, Urabe T. Cilostazol protects against brain white matter damage and cognitive impairment in a rat model of chronic cerebral hypoperfusion. Stroke. 2006; 37: 1539–1545. 10.1161/01.STR.0000221783.08037.a9 16645134

[19] Ye YL, Shi WZ, Zhang WP, Wang ML, Zhou Y, Fang SH, et al Cilostazol, a phosphodiesterase 3 inhibitor, protects mice against acute and late ischemic brain injuries. Eur J Pharmacol. 2007; 557: 23–31. 10.1016/j.ejphar.2006.11.003 17161838

[20] Ragab D, Abdallah DM, El-Abhar HS. Cilostazol renoprotective effect: Modulation of PPAR-γ, NGAL, Kim-1 and IL-18 underlies its novel effect in a model of ischemia-reperfusion. PLoS One. 2014; 9 10.1371/journal.pone.0095313 24816434 PMC4015937

[21] Gopinath K, Sudhandiran G. Protective effect of naringin on 3-nitropropionic acid-induced neurodegeneration through the modulation of matrix metalloproteinases and glial fibrillary acidic protein. Can J Physiol Pharmacol. 2016; 94: 65–71. 10.1139/cjpp-2015-0035 26544788

[22] Klein A, Wessolleck J, Papazoglou A, Metz GA, Nikkhah G. Walking pattern analysis after unilateral 6-OHDA lesion and transplantation of foetal dopaminergic progenitor cells in rats. Behav Brain Res. 2009; 199: 317–325. 10.1016/j.bbr.2008.12.007 19124044

[23] Glajch KE, Fleming SM, Surmeier DJ, Osten P. Sensorimotor assessment of the unilateral 6-hydroxydopamine mouse model of Parkinson’s disease. Behav Brain Res. 2012; 230: 309–316. 10.1016/j.bbr.2011.12.007 22178078 PMC3324279

[24] Yu-Taeger L, Petrasch-Parwez E, Osmand AP, Redensek A, Metzger S, Clemens LE, et al A Novel BACHD Transgenic Rat Exhibits Characteristic Neuropathological Features of Huntington Disease. J Neurosci. 2012; 32:15426 10.1523/JNEUROSCI.1148-12.2012 23115180 PMC6621569

[25] Rauch F, Schwabe K, Krauss JK. Effect of deep brain stimulation in the pedunculopontine nucleus on motor function in the rat 6-hydroxydopamine Parkinson model. Behav Brain Res. 2010; 210: 46–53. 10.1016/j.bbr.2010.02.003 20138919

[26] Fleming SM, Zhu C, Fernagut PO, Mehta A, DiCarlo CD, Seaman RL, et al Behavioral and immunohistochemical effects of chronic intravenous and subcutaneous infusions of varying doses of rotenone. Exp Neurol. 2004; 187: 418–429. 10.1016/j.expneurol.2004.01.023 15144868

[27] Cannon JR, Greenamyre JT. NeuN is not a reliable marker of dopamine neurons in rat substantia nigra. Neurosci Lett. 2009;464: 14–17. 10.1016/j.neulet.2009.08.023 19682546

[28] Wadie W, El-Tanbouly DM. Vinpocetine mitigates proteinuria and podocytes injury in a rat model of diabetic nephropathy. Eur J Pharmacol. 2017; 814 10.1016/j.ejphar.2017.08.027 28843828

[29] Bradford MM. A rapid and sensitive method for the quantitation of microgram quantities of protein using the principle of protein dye binding. Anal Biochem. 1976; 72: 248–254. 10.1016/0003-2697(76)90527-3 942051

[30] Zipp F, Aktas O. The brain as a target of inflammation: common pathways link inflammatory and neurodegenerative diseases. Trends Neurosci. 2006; 29: 518–527. 10.1016/j.tins.2006.07.006 16879881

[31] Amor S, Puentes F, Baker D, Van Der Valk P. Inflammation in neurodegenerative diseases. Immunology. 2010; 129:154–169. 10.1111/j.1365-2567.2009.03225.x 20561356 PMC2814458

[32] Kawai T, Takeuchi O, Fujita T, Inoue J, Muhlradt PF, Sato S, et al Lipopolysaccharide Stimulates the MyD88-Independent Pathway and Results in Activation of IFN-Regulatory Factor 3 and the Expression of a Subset of Lipopolysaccharide-Inducible Genes. J Immunol. 2001; 167: 5887–5894. 10.4049/jimmunol.167.10.5887 11698465

[33] Kaisho T, Akira S. Toll-like receptor function and signaling. J Allergy Clin Immunol. 2006; 117: 979–87; quiz 988. doi:S0091-6749(06)00439-8 [pii]\r 10.1016/j.jaci.2006.02.023 16675322

[34] Stefanova N, Reindl M, Neumann M, Kahle PJ, Poewe W, Wenning GK. Microglial activation mediates neurodegeneration related to oligodendroglial alpha-synucleinopathy: implications for multiple system atrophy. Mov Disord. 2007; 22: 2196–2203. 10.1002/mds.21671 17853477

[35] Okun E, Griffioen KJ, Lathia JD, Tang S-C, Mattson MP, Arumugam TV. Toll-like receptors in neurodegeneration [Review]. Brain Res Rev. 2009; 59: 278–292. 10.1016/j.brainresrev.2008.09.001 18822314 PMC2679904

[36] Perry VH, Cunningham C, Holmes C. Systemic infections and inflammation affect chronic neurodegeneration. Nat Rev Immunol. 2007; 7: 161–167. 10.1038/nri2015 17220915

[37] Tamandl D, Bahrami M, Wessner B, Weigel G, Ploder M, Furst W, et al Modulation of toll-like receptor 4 expression on human monocytes by tumor necrosis factor and interleukin-6: tumor necrosis factor evokes lipopolysaccharide hyporesponsiveness, whereas interleukin-6 enhances lipopolysaccharide activity. Shock. 2003; 20: 224–229. 10.1097/01.shk.0000079425.52617.db 12923493

[38] Park SY. Cilostazol Suppresses Superoxide Production and Expression of Adhesion Molecules in Human Endothelial Cells via Mediation of cAMP-Dependent Protein Kinase-Mediated Maxi-K Channel Activation. J Pharmacol Exp Ther. 2006; 317: 1238–1245. 10.1124/jpet.105.098509 16547169

[39] Park SY, Lee SW, Baek SH, Lee CW, Lee WS, Rhim BY, et al Suppression of PU.1-linked TLR4 expression by cilostazol with decrease of cytokine production in macrophages from patients with rheumatoid arthritis. Br J Pharmacol. 2013; 168: 1401–1411. 10.1111/bph.12021 23072581 PMC3596645

[40] Martin M, Rehani K, Jope RS, Michalek SM. Toll-like receptor-mediated cytokine production is differentially regulated by glycogen synthase kinase 3. Nat Immunol. 2005; 6: 777–784. 10.1038/ni1221 16007092 PMC1933525

[41] Hu X, Paik PK, Chen J, Yarilina A, Kockeritz L, Lu TT, et al IFN-γ Suppresses IL-10 Production and Synergizes with TLR2 by Regulating GSK3 and CREB/AP-1 Proteins. Immunity. 2006; 24: 563–574. 10.1016/j.immuni.2006.02.014 16713974

[42] Hong KW, Kim KY, Shin HK, Lee JH, Choi JM, Kwak Y-G, et al Cilostazol prevents tumor necrosis factor-alpha-induced cell death by suppression of phosphatase and tensin homolog deleted from chromosome 10 phosphorylation and activation of Akt/cyclic AMP response element-binding protein phosphorylation. J Pharmacol Exp Ther. 2003; 306: 1182–1190. 10.1124/jpet.103.052365 12807996

[43] Lee JH, Kim KY, Lee Y-K, Park SY, Kim CD, Lee WS, et al Cilostazol prevents focal cerebral ischemic injury by enhancing casein kinase 2 phosphorylation and suppression of phosphatase and tensin homolog deleted from chromosome 10 phosphorylation in rats. J Pharmacol Exp Ther. 2004; 308: 896–903. 10.1124/jpet.103.061853 14634032

[44] Zhao S, Fu J, Liu X, Wang T, Zhang J, Zhao Y. Activation of Akt/GSK-3beta/beta-catenin signaling pathway is involved in survival of neurons after traumatic brain injury in rats. Neurol Res. 2012; 34: 400–407. 10.1179/1743132812Y.0000000025 22643085

[45] Ma J, Wang Z, Liu C, Shen H, Chen Z, Yin J, et al Pramipexole-Induced Hypothermia Reduces Early Brain Injury via PI3K/AKT/GSK3β pathway in Subarachnoid Hemorrhage rats. Sci Rep. 2016; 6: 23817 10.1038/srep23817 27026509 PMC4812308

[46] Zhang W, He H, Song H, Zhao J, Li T, Wu L, et al Neuroprotective Effects of Salidroside in the MPTP Mouse Model of Parkinson’s Disease: Involvement of the PI3K/Akt/GSK3 β Pathway. Parkinsons Dis. 2016; 9450137 10.1155/2016/9450137 27738547 PMC5050371

[47] Jang M, Lee MJ, Kim CS, Cho IH. Korean red ginseng extract attenuates 3-nitropropionic acid-induced Huntington’s-like symptoms. Evidence-based Complement Altern Med. 2013; 2013: 237207 10.1155/2013/237207 23431333 PMC3568869

[48] Cross D a, Alessi DR, Cohen P, Andjelkovich M, Hemmings B a. Inhibition of glycogen synthase kinase-3 by insulin mediated by protein kinase B. Nature. 1995; 378: 785–789. 10.1038/378785a0 8524413

[49] Grimes CA, Jope RS. CREB DNA binding activity is inhibited by glycogen synthase kinase-3 beta and facilitated by lithium. J Neurochem. 2001; 78: 1219–1232. 10.1046/j.1471-4159.2001.00495.x 11579131 PMC1947002

[50] Park SY, Kim HY, Park HJ, Shin HK, Hong KW, Kim CD. Concurrent Treatment with Taxifolin and Cilostazol on the Lowering of β-Amyloid Accumulation and Neurotoxicity via the Suppression of P-JAK2/P-STAT3/NF-κB/BACE1 Signaling Pathways. PLoS One. 2016; 11 10.1371/journal.pone.0168286 27977755 PMC5158044

[51] Saraiva M, O’Garra A. The regulation of IL-10 production by immune cells. Nat Rev Immunol. 2010; 10: 170–181. 10.1038/nri2711 20154735

[52] Rangel-Barajas C, Coronel I, Florán B. Dopamine Receptors and Neurodegeneration. Aging Dis. 2015; 6: 349 doi: 10.14336/AD.2015.0330 26425390 10.14336/AD.2015.0330 PMC4567218

[53] Portera-Cailliau C, Hedreen JC, Price DL, Koliatsos VE. Evidence for apoptotic cell death in Huntington disease and excitotoxic animal models. J Neurosci. 1995; 15: 3775–3787. Available: http://www.jneurosci.org/content/15/5/3775%5Cnhttp://www.jneurosci.org/content/15/5/3775.full.pdf%5Cnhttp://www.jneurosci.org/content/15/5/3775.short%5Cnhttp://www.ncbi.nlm.nih.gov/pubmed/7751945 7751945 10.1523/JNEUROSCI.15-05-03775.1995 PMC6578226

[54] Keller JN, Guo Q, Holtsberg FW, Bruce-Keller AJ, Mattson MP. Increased sensitivity to mitochondrial toxin-induced apoptosis in neural cells expressing mutant presenilin-1 is linked to perturbed calcium homeostasis and enhanced oxyradical production. J Neurosci. 1998; 18: 4439–4450. 9614221 10.1523/JNEUROSCI.18-12-04439.1998 PMC6792705

[55] Tang S-C, Lathia JD, Selvaraj PK, Jo D-G, Mughal MR, Cheng A, et al Toll-like receptor-4 mediates neuronal apoptosis induced by amyloid beta-peptide and the membrane lipid peroxidation product 4-hydroxynonenal. Exp Neurol. 2008; 213: 114–21. 10.1016/j.expneurol.2008.05.014 18586243 PMC2597513

[56] Shin S, Le Lay J, Everett LJ, Gupta R, Rafiq K, Kaestner KH. CREB mediates the insulinotropic and anti-apoptotic effects of GLP-1 signaling in adult mouse β-cells. Mol Metab. 2014; 3: 803–812. 10.1016/j.molmet.2014.08.001 25379405 PMC4216406

[57] Satriotomo I, Bowen KK, Vemuganti R. JAK2 and STAT3 activation contributes to neuronal damage following transient focal cerebral ischemia. J Neurochem. 2006; 98: 1353–1368. 10.1111/j.1471-4159.2006.04051.x 16923154

[58] Yan Z, Gibson SA, Buckley JA, Qin H, Benveniste EN. Role of the JAK/STAT signaling pathway in regulation of innate immunity in neuroinflammatory diseases. Clin Immunol. 2018; 189:4–13. 10.1016/j.clim.2016.09.014 27713030 PMC5573639

[59] Cha B, Lim JW, Kim H. Jak1/Stat3 is an upstream signaling of NF-κB activation in Helicobacter pylori-induced IL-8 production in gastric epithelial AGS cells. Yonsei Med J. 2015; 56: 862–866. 10.3349/ymj.2015.56.3.862 25837197 PMC4397461

[60] Yu H, Pardoll D, Jove R. STATs in cancer inflammation and immunity: a leading role for STAT3. Nat Rev Cancer. 2009; 9: 798–809. 10.1038/nrc2734 19851315 PMC4856025

[61] Ozawa Y, Nakao K, Kurihara T, Shimazaki T, Shimmura S, Ishida S, et al Roles of STAT3/SOCS3 pathway in regulating the visual function and ubiquitin-proteasome-dependent degradation of rhodopsin during retinal inflammation. J Biol Chem. 2008; 283: 24561–24570. 10.1074/jbc.M802238200 18614536 PMC2528996

[62] Beurel E, Jope RS. Lipopolysaccharide-induced interleukin-6 production is controlled by glycogen synthase kinase-3 and STAT3 in the brain. J Neuroinflammation. 2009; 6: 9 10.1186/1742-2094-6-9 19284588 PMC2660311

[63] Beurel E, Jope RS. Differential regulation of STAT family members by glycogen synthase kinase-3. J Biol Chem. 2008; 283: 21934–21944. 10.1074/jbc.M802481200 18550525 PMC2494932

[64] Qin H, Buckley JA, Li X, Liu Y, Fox TH, Meares GP, et al Inhibition of the JAK/STAT Pathway Protects Against α-Synuclein-Induced Neuroinflammation and Dopaminergic Neurodegeneration. J Neurosci. 2016; 36: 5144–5159. 10.1523/JNEUROSCI.4658-15.2016 27147665 PMC6123006

[65] Shyu KG, Wang BW, Lin CM, Chang H. Cyclic stretch enhances the expression of toll-like receptor 4 gene in cultured cardiomyocytes via p38 MAP kinase and NF-kappaB pathway. J Biomed Sci. 2010; 17: 15 10.1186/1423-0127-17-15 20202224 PMC2844375

[66] Soliman A, Michelsen KS, Karahashi H, Lu J, Meng FJ, Qu X, et al Platelet-activating factor induces TLR4 expression in intestinal epithelial cells: implication for the pathogenesis of necrotizing enterocolitis. PLoS One. 2010; 5: e15044 10.1371/journal.pone.0015044 20976181 PMC2955554

[67] Gaudy AM, Clementi AH, Campbell JS, Smrcka AV., Mooney RA. Suppressor of cytokine signaling-3 is a glucagon-inducible inhibitor of PKA activity and gluconeogenic gene expression in hepatocytes. J Biol Chem. 2010; 285: 41356–41365. 10.1074/jbc.M110.159111 20978125 PMC3009861

[68] Yasukawa H, Ohishi M, Mori H, Murakami M, Chinen T, Aki D, et al IL-6 induces an anti-inflammatory response in the absence of SOCS3 in macrophages. Nat Immunol. 2003; 4: 551–556. 10.1038/ni938 12754507

[69] Dong Q, Fan R, Zhao S, Wang Y. Over-expression of SOCS-3 gene promotes IL-10 production by JEG-3 trophoblast cells. Placenta. 2009; 30:11–4. 10.1016/j.placenta.2008.10.008 19036437 PMC3066079

[70] Alexander WS. Suppressors of cytokine signaling (SOCS) in the immune system. Nat. Rev. Immunol. 2002; 2: 410–416. 10.1038/nri818 12093007

[71] Berlato C, Cassatella MA, Kinjyo I, Gatto L, Yoshimura A, Bazzoni F. Involvement of Suppressor of Cytokine Signaling-3 as a Mediator of the Inhibitory Effects of IL-10 on Lipopolysaccharide-Induced Macrophage Activation. J Immunol. 2002; 168: 6404–6411. 10.4049/jimmunol.168.12.6404 12055259

[72] Antoniv TT, Ivashkiv LB. Interleukin-10-induced gene expression and suppressive function are selectively modulated by the PI3K-Akt-GSK3 pathway. Immunology. 2011; 132: 567–577. 10.1111/j.1365-2567.2010.03402.x 21255011 PMC3075510

